# Comment on: What is new in the treatment of snakebite envenoming? Opportunities and challenges

**DOI:** 10.1093/trstmh/traf023

**Published:** 2025-02-26

**Authors:** Andreas H Laustsen

**Affiliations:** Department of Biotechnology and Biomedicine, Technical University of Denmark, Søltofts Plads 224, DK-2800 Kongens Lyngby, Denmark

Improving treatment for the millions of patients that fall victim to snakebite envenoming each year remains a pressing issue, and it was with great pleasure that I read Professor Warrell's editorial on the opportunities and challenges that exist around this challenge.^1^ In particular, I appreciate Professor Warrell’s views around clinical trials and suggestions on how these can be conducted in a pragmatic and (financially) feasible manner. However, while I share Professor Warrell's concerns around the value of many of the preclinical models in regard to how well they translate to human envenoming cases, I feel obliged to share my views on the area of recombinant antivenoms, where I believe Professor Warrell's scepticism should perhaps be replaced by cautious optimism in view of recent developments.

Professor Warrell shares some excitement around the recent discovery of a human monoclonal antibody and its utility in neutralizing long-chain α-neurotoxins from elapids.^2^ This achievement by Khalek et al. is indeed impressive, but I would like to bring to Professor Warrell's and other's attention that this discovery is no lonely swallow; broadly neutralizing monoclonal antibodies and nanobodies have now been reported in multiple instances, both before and after the report from Khalek et al.^3–6^ Moreover, the idea of manufacturing oligoclonal mixtures of monoclonal antibodies is now no longer purely theoretical, but has recently been demonstrated to be feasible.^7^ Finally, it has also been shown that surprisingly simple oligoclonal mixtures of nanobodies can be used to neutralize coral snake venoms and outperform a clinically approved coral snake antivenom in regards to potency and cross-neutralization capacity.^6^ Combined with recent reports demonstrating how novel *in silico*–based discovery methods can also be used to discover effective toxin-neutralizing proteins in a rapid manner, even without access to physical venoms or toxins,^8^ I would argue that the development of recombinant antivenoms is now technologically within reach.

Professor Warrell rightly states that snake venoms are exceptionally complex and illustrates this with three examples of venoms from South Asian Russell's vipers, Lesser Antillean pit vipers and South Asian kraits. He further raises the point that toxicovenomics cannot be blindly used to assess or guide the development of new antivenom products. While I do not disagree with Professor Warrell, I would like to argue two points. First, while toxicovenomics might not be the perfect navigation system, it does serve as a useful guiding principle—perhaps comparable to a traditional map and compass. In the absence of better systems to navigate venom complexity, we must use what is available, until better methodologies are developed. Second, I want to argue that the use of recombinant DNA technology might be what is needed to deal with venom complexity, as recombinant expression enables the antivenom manufacturer to design and optimize the composition of a recombinant antivenom to match the venoms it is indicated for. So, while I fully support the notion that new products must be evaluated in more robust preclinical models accounting for the complex pathogeneses induced by snake venoms, as well as adequately designed clinical trials to demonstrate translatability to the human setting, we must not let the current lack of knowledge within toxinology hinder the introduction of new innovations with the potential to overcome the existing drawbacks (safety, efficacy, cost of manufacture) of polyclonal antivenoms derived from immunized animals.

## Data Availability

N/A.
